# Intelligence

**Published:** 1807-05

**Authors:** 


### INTELLIGENCE.

Mr. Cornelius Varley has laid before the public some remarks on atmospherical phenomena, particularly on the formation of clouds; their performance; their precipitation in rain, snow, and hail; and the consequent rise of the barometer. The inferences drawn by this gentleman are, 1. That no cloud can be formed, or exist without electricity. 2. That no cloud can fall in rain till it parts with some of its electricity. 3. That in fine weather the earth must be giving electricity to the atmosphere by means of vapour, and in stormy weather, the atmosphere must be giving electricity in the earth by means of vapour, rain, or lightning. 4. That in fine weather the clouds are separating, and in stormy weather uniting. 5. That electricity is the suspending power in clouds. 6. That dry air is a conductor of heat, but a non-conductor of electricity. 7. That water can exist permanently in four states, and temporarily in one only. Two of these are effected by electricity, and three without it. The first electrical state is that of cloud, which is so much charged as to become lighter than air at the surface of the earth; the second is a complete saturation of water with the electric fluid, which produces a transparent and elastic fluid light enough to float above the highest clouds. The first of the three other states is ice; the second is liquid; the third, which is quite temporary, is vapour; for, as soon as the supply of heat by which it is raised from the earth is withdrawn, it condenses, and returns again to the state of water. A consequence of this theory is, that when a cloud loses its electricity in an atmosphere below the freezing point, then snow is produced, for the vapours will be frozen in the act of uniting: and particles of moisture united into rain, and passing through a cold region in their descent to the earth, will come down in the form of hail.

M. Tenon has lately presented to the National Institute of France, a description of the cahalot and crocodile. The teeth of the former have no enamel, but only the osseous cortex. The one, we are informed, may be easily distinguished from the other, because the enamel is much harder, and is entirely dissolved in the acids, without leaving any gelatinous parenchyme. The trunks of the elephant, and the grinders of the bear, have no other envelope. The same able anatomist, is about to publish an important work work on the eye, and the diseases to which it is subject. He has made several new remarks upon the parts which surround this organ: he has found some tendinous lumps which tie the straight muscles to the anterior edges of the orbit, and serve them for a kind of returning pulley, and hinder them from compressing the eye-ball: he has developed a membranous tunic which surrounds the eye-ball, attaches it to the two angles of the orbit by two kinds of wings, passes into the pupils, and is there reflected behind the tarsi, and gives a passage to the tendons of the muscles: he has established a new opinion upon the agents which transmit to the iris the action of the retina, and by which, the impressions received by the latter, dilate or contract the other; these agents he finds in the ciliary processes, the tongues of which are prolonged behind the iris, and the tails of them touch the retina. M. Tenon has also discovered that the hare-lip sometimes proceeds from a rent of one of the maxillary bones, sometimes from a rent in both; and he attributes the cause of it to a disproportionaté dilatation of the tongue. He asserts that it is highly dangerous to perform any operation for the hare-lip at the time when the teeth are cutting.

The mineral waters of Lipetzk, in the province of Tambow, in Russia, have lately been analysed by Mr. Skell, and are found to contain in one pound as follows:

| | |
|---|---|
| Carbonat of iron - - - | $\frac{29}{16}$ grains |
| ——— of lime - - - | $\frac{3}{86}$ |
| Muriate of magnesia - - - | $\frac{8}{25}$ |
| ——— of soda - - - - | $2\frac{1}{12}$ |
| Sulphate of lime - - - | $\frac{1}{8}$ |
| ——— of soda - - - | $\frac{1}{2}$ nearly |
| Bitumen - - - - - - | $\frac{7}{100}$ |

From these analyses, and other accurate observations, it should seem that the water at Lipetzk has some analogy to that of Pyrmont: it, has, however, less of the irritating quality, with regard to the carbonic; less of the power of solution with respect to salts, and more of the tonic powers of iron. On these accounts Mr. S. asserts that the water of Lipetzk stimulates, gives vigour, increases the elasticity of the muscular fibres and the activity of the organs, enriches the blood, and imparts more colour to it; while on the other hand it liquifies tenacious, slimy, and condensed fluids, removes obstructions in the canals, qualifies the sharpness of humours, and destroys worms.

The Prize given by the Royal College of Surgeons, London, for the best Dissertation on Hernia, for the year 1806, has been adjudged to Mr. LAWRENCE, of John Street, Adelphia. And the Prize for the best Dissertation on the Diseases of the Joints, to Mr. S. COOPER, of Golden Square.

We have in our number for February, already noticed a new theory, advanced by Dr. WOLLASTON, and cited by Mr. Davy, in his Lectures of the Fairy Rings. A paper from Dr. Wollaston on this subject has been read to the Royal Society, of which we shall hereafter give a more detailed account.

Mr. CARPUE will commence his Summer Course of Lectures on Anatomy and Surgery, the first Monday in June. Particulars may be known by applying to Mr. Carpue, No. 50, Dean-street, Soho.

Mr. JOHN TAUNTON, Member of the Royal College of Surgeons in London, Surgeon to the City and Finsbury Dispensaries, &c. will commence his Lectures on Anatomy and Surgery, on Saturday, May 30, 1807, at No. 21, Greville-street, Hatton-garden.

Dr. WALKER is preparing for the press, an Essay on Vaccination, with some Account of its Rise and Progress, of the Authors who first promulgated and established the Practice, and of the Associations under the Titles of Original Vaccine Pock Institution, Royal Jennerian Society, and London Vaccine Institution, formed in the Metropolis for its further propagation.

---

*Poisonous Effects of the Effluvia of Sumach on a Swarm of Bees:* By JAMES SOMERVILLE, Esq. of West-Chester County, N. America.

SUPPOSING that the qualities of plants and their effects on animal life are generally interesting, and that any new facts concerning them will be acceptable, I am induced to communicate the following fact concerning the *rhus vernix*, Linn. commonly called *swamp-sumach*, from a memorandum I made at the time of the occurrence, but which had been mislaid or neglected till a late conversation on that subject led me to look it up.

On the 21st of last June, a neighbour of mine had a swarm of bees, which he found attached to a branch of the above-mentioned shrub. A box made of common pine was put over them in the usual manner: to effect which, a bench was placed near, and the branch bent down to it, and in part cut off; this was 3 o'clock in the afternoon; about 9 o'clock the same evening they were removed to the place where it was intended they should remain; next morning, about 5 o'clock, they were all found *dead*, with very few exceptions, and these in a state of torpor and extreme debility, which, in a short time after being exposed to the fresh air, ended also in death. They appeared much swelled beyond the ordinary size, perhaps a third; their colour uncommonly black. The swarm was the first for the season from that stock, and seemed to be a very large one. The box was inspected and found to be free from any thing of an offensive or obnoxious nature, and has since been used for another swarm.

The deleterious qualities of this plant have been frequently experienced by an incautious approach to it in its growing state; and also when used as fuel along with other wood, it emits a smoke very injurious; but I have never known so strong an instance of its power as that above related, being equal to sulphur in its effects on the bees.

From what is known of this plant and some others, the wonders related related of the Bohun-upas, of Java, are rendered somewhat less incredible; and, perhaps, are only an account of facts exaggerated by the oriental manner of description.

---

HOSPITAL FOR THE MAINTENANCE AND EDUCATION OF EXPOSED AND DESERTED YOUNG CHILDREN.

"At a General Committee, Wednesday, March 18, 1807,— read the following Report from the Physicians and Surgeon of this Hospital:—

"Dr. Mayo, Dr. Stanger, and Mr. Ramsden, report to the Committee of the Foundling Hospital, that twenty-one of the children who were vaccinated on the 10th of April, 1801, and inoculated with small-pox matter on the 9th of August, 1802, and again on the 13th of November, 1804, were re-inoculated with small-pox matter on the 23d of February, 1807, without any consequence; except slight inflammation of the inoculated part in a few instances, and in three cases, a small pustule on the part where the matter was inserted.—

"RESOLVED, That the Secretary transmit a Copy of the above Report to the College of Physicians, and to the Secretary of the Jennerian Society.

( "Extract from the Minutes. )

"MORRIS LIVESLEY, Secretary."

